# The impact of ambient temperature on mortality among the urban population in Skopje, Macedonia during the period 1996–2000

**DOI:** 10.1186/1471-2458-6-44

**Published:** 2006-02-23

**Authors:** Vladimir T Kendrovski

**Affiliations:** 1 Department for Hygiene and Environmental Health, Medical Faculty, University "St. Cyril and Methodius", 50 Divizija 6, Skopje, Republic of Macedonia

## Abstract

**Background:**

This study assesses the relationship between daily numbers of deaths and variations in ambient temperature within the city of Skopje, R. Macedonia.

**Methods:**

The daily number of deaths from all causes, during the period 1996–2000, as well as those deaths from cardiovascular diseases, occurring within the city of Skopje were related to the average daily temperature on the same day using Multiple Regression statistical analyses. Temperature was measured within the regression model as two complementary variables: 'Warm' and 'Cold'. Excess winter mortality was calculated as winter deaths (deaths occurring in December to March) minus the average of non-winter deaths (April to July of the current year and August to November of the previous year).

**Results:**

In this study the average daily total of deaths was 7% and 13% greater in the cold when compared to the whole period and warm period respectively. The same relationship was noticed for deaths caused by cardiovascular diseases. The Regression Beta Coefficient (b = -0.19) for the total mortality as a function of the temperature in Skopje during the period 1996–2000 was statistically significant with negative connotation as was the circulatory mortality due to average temperature (statistically significant regression Beta coefficient (b = -0.24)). A measure of this increase is provided, on an annual basis, in the form of the excess winter mortality figure.

**Conclusion:**

Mortality with in the city of Skopje displayed a marked seasonality, with peaks in the winter and relative troughs in the summer.

## Background

All climate and weather variables have some influence on human health. The effect may be either directly on the human body or indirectly through effects on disease-causing organisms or their vectors. Although the effects of variation of only one weather element may be examined in a particular study, that element does not act independently of other elements, for example, changes in humidity modify the effects of temperature. An association between cardiovascular deaths and outdoor temperature has been a topic of interest since 1970 [1-4]. It is generally agreed that as temperature decreases the risk of death increases. Increases in coronary events have also been seen during heat waves. Interactions between climate and health are location-specific; using epidemiological evidence based on local data if they are available is therefore important. It is well-known that urban areas experience specialized problems relating to climate and human health [5]. For example, heat-related mortality is excessive in these areas, and pollution problems may exacerbate other conditions, such as asthma and respiratory distress. Recent studies in Europe and Russia show that mortality increases to a greater extent for a given fall in temperature in regions such as the UK and Greece that have relatively warm winters, than in colder countries where priority is given to keeping warm regardless of other factors. This was associated with cooler homes and wearing of less effective clothing outdoors in countries with mild winters [6-8].

Although Republic of Macedonia is relatively small country, its territory is covered by different types of climate: from continental, changed continental, sub-Mediterranean (changed maritime) to mountainous climate with various subtypes. Within the territory of the Republic of Macedonia the influences of the Mediterranean and the continent overlap, with different spectrum of influences. Macedonia has a basic Mediterranean climate of hot, dry summers; winters, however, are somewhat warmer than in northern neighboring countries due to the influence of the winds from the Aegean Sea. Average daily temperature extremes in Skopje range from 32°C to -3°C. The summers are warm, dry and long lasting; and the winters are cold, wet with foggy and snowy days [9].

The main goal of this study is to increase awareness about the impact of climatic on health in a densely populated area in Macedonia by relating mortality data to outside air temperature in the urban area of Skopje during the period 1996 to 2000.

## Methods

The study was carried out in the city of Skopje, Republic of Macedonia. Its population, according to the census of 2002, is over 500.000 inhabitants. The epidemiological study is designed as retrospective research. The research material is presented as climatic data (the ambient air: daily average temperature) as well as mortality daily data for Skopje as urban area. Quantifying temperature-related mortality for Skopje requires access to daily mortality data as well as daily climatic (air temperature) data. The daily number of deaths in Skopje was obtained from the State Statistical Office, Mortality Register and was restricted to city residents only. The completeness of the register, the quality of patient diagnosis and certificated cause of death have been examined and it has been demonstrated that the register is both complete and reliable, i.e. more than 99% of deaths are confirmed by physicians. The causal groups considered in this study are total mortality and deaths from diseases of the circulatory system (ICD-9 codes 390–459). The mean daily temperature for Skopje (city's weather station) was obtained from the National Hydro-meteorological Institute.

The large number of days (N = 1827) for the study period from 01.01.1996 to 31.12.2000, i.e. the distribution of daily total deaths in Skopje, make possible the stratification of the sample in two variables ("warm" and "cold") in order to assess the effect of ambient temperature on mortality in different months without big fluctuation in temperature values. In each, analysis was carried out for the entire period of study and for two semestral periods. (Table 1) Specifically, it was decided to group the relatively colder months (October-April), and the hotter months (May-October). The historical long-term monthly temperature average data for Skopje showed that April is colder than October.

In this article we only present the results of the stratified analysis since this more reflects the dynamic relationship between temperature and mortality.

The daily number of deaths from all causes, during the period 1996–2000, as well as those deaths from cardiovascular diseases, occurring within the city of Skopje were related to the average daily temperature on same day using Multiple Regression statistical analyses. Temperature was measured within the regression model as two complementary variables: 'Warm' and 'Cold'. Scatter charts for mortality residuals data were produced for total deaths and temperature for all variables

Excess winter mortality is calculated as winter deaths (deaths occurring in December to March) minus the average of non-winter deaths (April to July of the current year and August to November of the previous year) [10].

## Results

The mean daily number of deaths among the citizens of the city of Skopje for 1996–2000 were 10.01 with a standard deviation of 3.45. (Table 2)

In this study average daily total of deaths was 7% and 13% greater in the cold when compared to the whole period and warm period respectively. The same relation was noticed for deaths caused by cardiovascular diseases.

**Figure 1:**
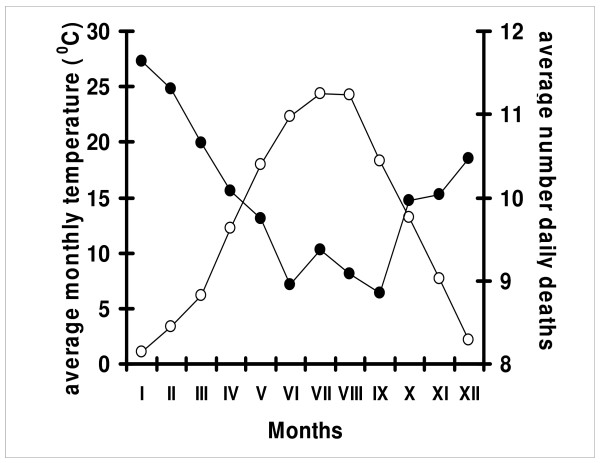
The distribution of average daily number of deaths from all causes for the period 1996–2000 in Skopje by months.

Also we noticed that January, February, March and December were months with greater daily mortality among the citizens of Skopje for the estimated period when compared to the summer months: September, June, August and July. (Figure 1)

**Figure 2:**
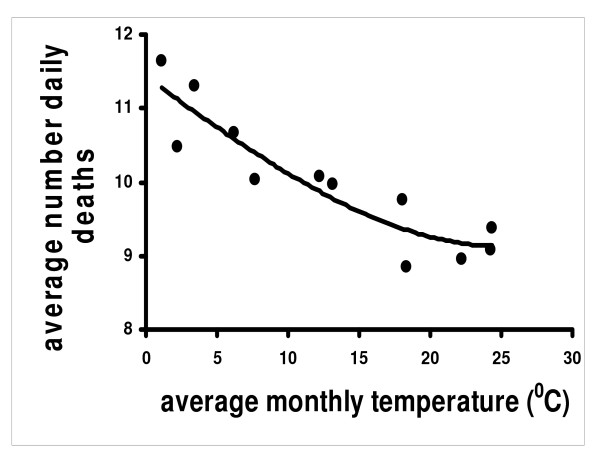
The relationship between average daily number deaths from all causes and average monthly temperature for the period 1996–2000.

From a graphical analysis of the whole period, a relationship between the average daily deaths from all causes and average monthly temperature appears graphically as a polynomial quadratic equation (Figure 2).

The warmest month was July with average monthly temperature of 24,34°C and January was the coldest month with 1,1°C average monthly temperature, retrospectively. The January value is 16.3% greater than the yearly average daily number of deaths (10.01), and the September value 11.6% below the yearly average. The difference between January and September is 27.9%. (from +16.3 to -11.6)

The nature of the relationship between temperature and mortality was also analysed by season consistent with those presented here. The Regression Beta Coefficient (b = -0.19) for the total mortality as function of the temperature in Skopje during the period 1996–2000 as well as was for the daily average mortality from diseases of the circulatory system (b = -0.24) were statistically significant with negative connotation.

Mortality within the city of Skopje displayed a marked seasonality, with peaks in the winter and relative troughs in the summer.

**Figure 3:**
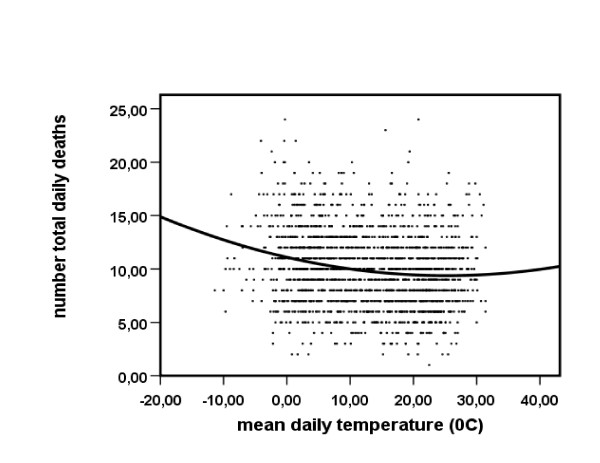
Scatter diagram for all cause daily mortality and mean daily temperature, Skopje, 1996–2000.

From a graphical analysis of the whole period, a relationship between the number of daily deaths from all causes and mean daily temperature appears graphically as a nonmonometric 'U' shape – the lowest mortality rate being reached at temperatures within a range between 17–23°C. (Figure 3)

**Figure 4:**
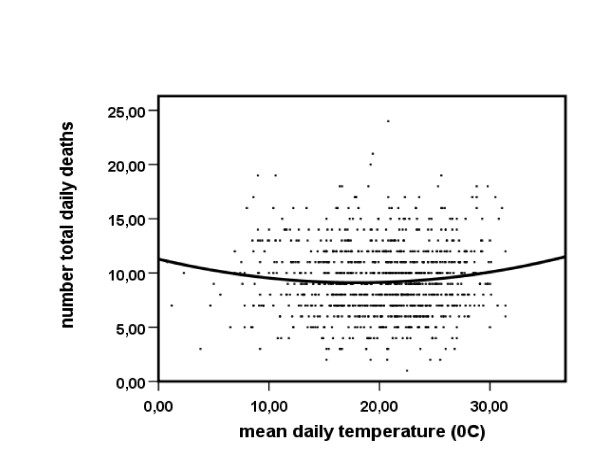
Scatter diagram for all cause daily mortality and mean daily temperature, Skopje, warm months, 1996–2000.

Taking the two periods separately, it can be seen that, as well as a seasonal effect (i.e. more deaths occurring during the 'cold' months than in the 'warm' months), the affect of temperature on all cause daily mortality rate is much clearer when mortality rate is related to either the 6 coldest or the 6 warmest months of the year (Figure 3 and Figure 4) rather than to the whole year.

The mortality minima can be found within a wide temperature range centred around 14°C during the colder months and around 21°C during the hotter month. In order to assess mortality levels over the winter as a whole we estimated the excess winter mortality for four winters in the period 1996–2000 for Skopje. (Table 3)

The results show that winter mortality varied from winter to winter, being highest in the winter of 1999/2000, accounting for 47% of all excess deaths of the combined four winters examined.

## Discussion

In this study, the air temperature at which all cause mortality reached its minimum was around 18°C. This value is lower to that found in other studies carried out in lower zones with latitudes and climate similar to Skopje e.g. Valencia, Athens and Barcelona with around 24°C [11-13]. Nevertheless, in other areas this minimum is located around manifestly different temperatures. In a subtropical country such as Taiwan it is situated around 26–29°C whereas in a country such as Holland, with its colder and more humid climate, it is around 16.5°C [14,15]. Two hypotheses have been advanced to explain these differences: on the one hand, they could reflect differences in housing conditions (insulation, heating etc.) or differences in lifestyle patterns, with people living in warmer countries spending longer outdoors. On the other hand, it could also correspond to an acclimatization process to the variations in temperature. Respiratory infections may be responsible for deaths whose basic cause has been classified as cardiovascular. An indirect mechanism which may help to account for this process would be the increase in platelet aggregation during infections [15,16].

In this study average daily of total deaths is 7% and 13% greater in the cold months compared to the whole period and hot period respectively. The size of the increased risk attributable to cold temperatures seems to depend on the average annual temperature; for example, the Eurowinter group found smaller increases in cardiovascular mortality in colder regions (Finland) than in warmer regions [4]. Increased mortality from coronary thrombosis in the cold is associated with haemoconcentration (thickening of the blood), which occurs as fluid is lost from the circulation after vasoconstriction in the cold, and with increased blood pressure and with raised fibrinogen levels due to respiratory infections in winter. Arterial thrombosis due to these changes causes just over half of the excess winter deaths, and respiratory disease approximately a further 20%. A hypothesised pathway for the increased risk of coronary events in colder periods is that exposure to cold temperatures triggers rapid release of cathecholamines with consequent rises in vasomotor tone, haemodynamic parameters, platelet aggregability, and other haemathological and endothelial parameters [17]. When a person comes from warm indoor to cold conditions, the subsequent increase in cardiac workload and oxygen consumption may trigger an acute coronary syndrome [18-22].

Excess winter mortality has been reported in medical journals for about 150 years, and most countries suffer from 5% to 30% excess winter mortality [23]. However, there still remains much debate with regard to why certain countries experience dramatically higher rates of seasonal mortality than others. In this study the winter of 1999/2000, accounting for 47% of all excess deaths of the combined four winters examined. We need additional investigation to discuss what was unique in this winter to account for 2,5 times the expected number of deaths (47%). Cold strain from both indoors and outdoors has been implicated on several occasions, however other potential factors (other than cold strain) have rarely been analysed. Besides factors associated with biological and genetic considerations that have been linked with reduced health status, the health of a population is influenced by a large number of factors. In this study women showed greater winter mortality in cold periods compared with men. However, there are also physiological sex differences in thermoregulation, including longer recovery time to body temperature after physical exercise among women, which could partly mediate the increased risks in women [24].

## Conclusion

There are a number of limitations due mainly to the ecological design of the study. Another weakness is that we did not control for air pollution, and increases in air pollution have been shown to correlate with the risk of a cardiovascular event, and the seasonal change in risk. Also we were not able to control for respiratory infections, which are common in cold and damp conditions and may precipitate a coronary event.

In this study it has been shown that mortality rates in the urban area of Skopje are increased during the winter months. It is suggested that the excess is preventable and is most probably attributable to poor protective measures taken on cold days or other related factors. It is concluded that efforts should be made to make the general public more aware about the phenomena of excess winter mortality, particularly in Macedonia.

## Competing interests

The author(s) declare that they have no competing interests.

## Pre-publication history

The pre-publication history for this paper can be accessed here:



## Figures and Tables

**Figure 5:**
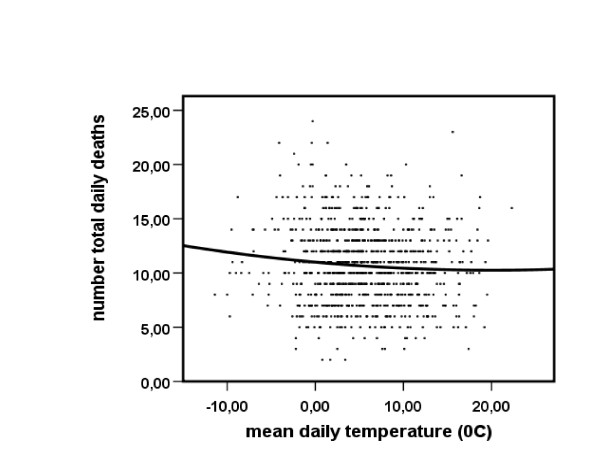
Scatter diagram for all cause daily mortality and mean daily temperature, Skopje, cold months, 1996–2000.

**Table 1:** The design of the Study- distribution by variables

*Study period: from 01.01.1996 to 31.12.2000 *VARIABLE: **TOTAL N(days) = 1827**	
VARIABLE: **COLD MONTHS IN 1996–2000 ***(JANUARY, FEBRUARY, MARCH, APRIL, NOVEMBER, DECEMBER) ***N(days) = 907**	VARIABLE: **WARM MONTHS IN 1996–2000 ***(MAY, JUNE, JULY, AUGUST, SEPTEMBER, OCTOMBER) ***N(days) = 920**

**Table 2:** The distribution of daily average mortality (total and specific mortality data) and temperature in Skopje for the period 1996–2000 by variables

	***TOTAL (1996–2000)***				***WARM MONTHS***		***COLD MONTHS***	
	
		***SD***	*min*.			***SD***		***SD***
Total mortality	10,01	3,45	1,00	24,00	9,33	3,25	10,69	3,52
Mortality from circulatory diseases	5,38	2,46	1,00	17,00	4,85	2,26	5,91	2,53
Temperature (°C)	12,80	9,08	-11,40	31,40	20,02	5,42	5,41	5,42

**Table 3:** The excess winter mortality in Skopje during the period 1996–2000

	**Excess winter mortality in absolute numbers**			**Index of Excess winter mortality in %**		
**Winter**	**Males**	**Females**	**Total**	**Males**	**Females**	**Total**

**1996/1997**	110,5	89	199,5	**18**	**18**	**18**
**1997/1998**	12	58,5	70,5	**1**	11	**6**
**1998/1999**	45	69	114	**6,6**	13,1	**9,5**
**1999/2000**	130,5	213,5	344	19,7	44,1	**30**
**Total**	298	430	728	**11.3**	21.5	15,9
